# Cooperation between Apoptotic and Viable Metacyclics Enhances the Pathogenesis of Leishmaniasis

**DOI:** 10.1371/journal.pone.0005733

**Published:** 2009-05-29

**Authors:** João Luiz Mendes Wanderley, Lucia Helena Pinto da Silva, Poliana Deolindo, Lynn Soong, Valéria Matos Borges, Deboraci Brito Prates, Ana Paula Almeida de Souza, Aldina Barral, José Mario de Freitas Balanco, Michelle Tanny Cunha do Nascimento, Elvira Maria Saraiva, Marcello André Barcinski

**Affiliations:** 1 Experimental Medicine Division, National Cancer Institute, Rio de Janeiro, Rio de Janeiro, Brazil; 2 Morphological Sciences Program, Biomedical Sciences Institute, Federal University of Rio de Janeiro, Rio de Janeiro, Brazil; 3 Department of Microbiology and Veterinary Immunology, Veterinary Institute, Federal Rural University of Rio de Janeiro, Rio de Janeiro, Rio de Janeiro, Brazil; 4 Molecular and Cellular Biology Program, Oswaldo Cruz Foundation, Rio de Janeiro, Rio de Janeiro, Brazil; 5 Departments of Microbiology and Immunology and of Pathology, Center for Biodefense and Emerging Infectious Diseases, Sealy Center for Vaccine Development, University of Texas Medical Branch, Galveston, Texas, United States of America; 6 Gonçalo Muniz Research Center, Oswaldo Cruz Foundation, Salvador, Bahia, Brazil; 7 Immunology Department, Microbiology Institute, Federal University of Rio de Janeiro, Rio de Janeiro, Rio de Janeiro, Brazil; 8 Parasitology Department, Biomedical Sciences Institute, University of Sao Paulo, Sao Paulo, Sao Paulo, Brazil; Federal University of São Paulo, Brazil

## Abstract

Mimicking mammalian apoptotic cells by exposing phosphatidylserine (PS) is a strategy used by virus and parasitic protozoa to escape host protective inflammatory responses. With *Leishmania amazonensis* (*La*), apoptotic mimicry is a prerogative of the intramacrophagic amastigote form of the parasite and is modulated by the host. Now we show that differently from what happens with amastigotes, promastigotes exposing PS are non-viable, non-infective cells, undergoing apoptotic death. As part of the normal metacyclogenic process occurring in axenic cultures and in the gut of sand fly vectors, a sub-population of metacyclic promastigotes exposes PS. Apoptotic death of the purified PS-positive (PS^POS^) sub-population was confirmed by TUNEL staining and DNA laddering. Transmission electron microscopy revealed morphological alterations in PS^POS^ metacyclics such as DNA condensation, cytoplasm degradation and mitochondrion and kinetoplast destruction, both in *in vitro* cultures and in sand fly guts. TUNEL^POS^ promastigotes were detected only in the anterior midgut to foregut boundary of infected sand flies. Interestingly, caspase inhibitors modulated parasite death and PS exposure, when added to parasite cultures in a specific time window. Efficient *in vitro* macrophage infections and *in vivo* lesions only occur when PS^POS^ and PS-negative (PS^NEG^) parasites were simultaneously added to the cell culture or inoculated in the mammalian host. The viable PS^NEG^ promastigote was the infective form, as shown by following the fate of fluorescently labeled parasites, while the PS^POS^ apoptotic sub-population inhibited host macrophage inflammatory response. PS exposure and macrophage inhibition by a subpopulation of promastigotes is a different mechanism than the one previously described with amastigotes, where the entire population exposes PS. Both mechanisms co-exist and play a role in the transmission and development of the disease in case of infection by *La*. Since both processes confer selective advantages to the infective microorganism they justify the occurrence of apoptotic features in a unicellular pathogen.

## Introduction

Programmed cell death by apoptosis plays a central role in normal tissue development and homeostasis. The process of removal of apoptotic cells by neighboring phagocytes includes efficient engulfment of the dying cells prior to the leakage of their plasma membrane, as well as the induction of synthesis and release of anti-inflammatory mediators. Both mechanisms contribute to suppression of an inflammatory reaction in the micro-environment surrounding apoptotic cells [1], [2]. Clearly, multicellular organisms can benefit from death by apoptosis of developmentally unnecessary, infected or potentially harmful sub-populations of their own cells [3]. By contrast, the reason why apoptotic death occurs in unicellular organisms is not easily conceivable. However, different forms of programmed cell death, with features identical or similar to apoptotic death, have been described in at least nine different species of unicellular eukaryotes belonging to four different branches of the phylogenetic tree [4]. It is worth noting that most of the species on which such forms of cell death were described are mammalian parasites, agents of important tropical diseases such as leishmaniasis [5], [6], African and South American trypanosomiasis [7], [8], [9], and malaria [10], transmitted by insect vectors and thus having to cope with environmental transitions characteristic of a digenetic organism. Leishmanial diseases are transmitted by sand fly vectors, which inoculate flagellated promastigotes into a mammalian host. Promastigotes differentiate into amastigotes inside phagolysosomes and the disease progresses due to sequential macrophage infection with amastigotes [11]. In an infection with *La*, amastigote forms display PS on their external membrane outer leaflet, a typical apoptotic feature of multicellular organisms without necessarily leading to parasite death. As a result of PS recognition, leishmanial intracellular survival is ensured due to host phagocyte inactivation [12], [13]; this phenomenon has been named apoptotic mimicry [12]. A very similar situation has recently been described with vaccinia virus [14], characterizing apoptotic mimicry as a more general phenomenon of escape from host inflammatory response. As we have shown, the host modulates PS exposure by amastigotes and, as a consequence, parasites derived from susceptible BALB/c mice display a significantly higher density of PS moieties than parasites derived from less susceptible C57BL6 mice. The density of PS molecules on the surface of the amastigotes defines their ability to be internalized and to inhibit macrophage inflammatory capacity [13]. Now we show that programmed apoptotic death of a sub-population of promastigotes is part of the developmental changes occurring during normal metacyclogenesis, the process by which promastigotes evolve from a non-infective (procyclic) to a fully infective (metacyclic) form [15]. As a consequence of the programmed death during metacyclogenesis, the population of metacyclic promastigotes of *La*, derived from cultures or from the gut of the insect vector, is composed of PS^POS^ and PS^NEG^ forms. PS^POS^ population displays ultrastructural and biochemical features of apoptotic death, while the PS^NEG^ population is the truly infective one. Both forms, when purified, are able to internalize into macrophages; however, intracellular multiplication of PS^NEG^ forms only occurs when the phagocytes are infected in the presence of PS^POS^ forms, which are capable of inhibiting production of nitric oxide by activated macrophages. This mechanism of cooperation between different subpopulations of promastigotes also operates *in vivo*. Our present results confirm and extend the report showing that the presence of apoptotic promastigotes in the virulent inoculum is important for the development of the experimental disease with *L. major* [16]. The cell biology of PS exposure in promastigotes, which occurs via apoptotic death, differs from the one in amastigotes occurring via apoptotic mimicry [12]. They take place at different stages of the disease caused by *La*: the first one is required at the moment of infection, and the second one, for disease progression in the mammalian host. They emphasize the necessity of surface PS for the generation of a permissive host for the survival and proliferation of *La*.

## Results

### A sub-population of metacyclic promastigotes from *in vitro* cultures or from the sand fly gut displays PS on its surface

**Figure 1:**
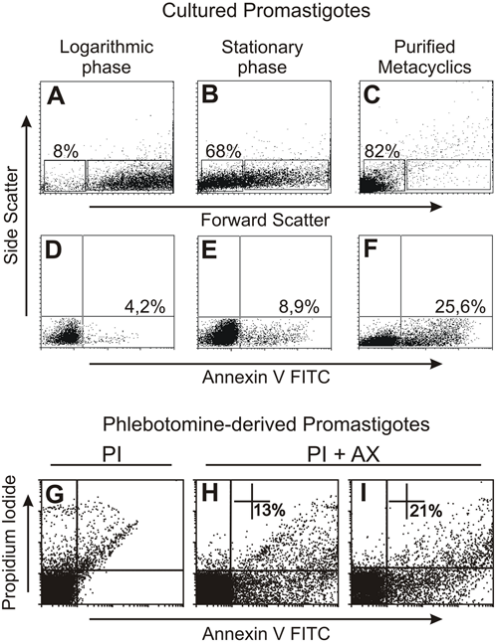
A fraction of metacyclic promastigotes exposes phosphatidylserine *in vivo* and *in vitro*. Characterization of small size (R2-lower left gate) and large size (R1-lower right gate) sub-populations of (A) logarithmic, (B) total stationary, and (C) purified metacyclic promastigotes, showed by dot-plots of forward light scatter (FSC) vs. side angle light scatter (SSC) obtained by flow cytometric analysis. PS exposure, assessed by AnV staining in (D) logarithmic, (E) total stationary, and (F) infective metacyclic populations. Figure displays one representative experiment out of five with similar results. PS exposure in promastigotes purified from the gut of *Lutzomya longipalpis* at (H) 5 days and (I) 9 days after artificial feeding with J774 cells infected with *La* and (G) the appropriate control parasites stained with PI only. Figure displays one experiment out of two with similar results.

To compare the amount of PS exposure by logarithmic and stationary-phase promastigotes, parasites obtained from 2 day-old cultures after at least 3 short-term consecutive passages (logarithmic), and from 6 to 7 day-old cultures (stationary), as well as a population enriched for infective metacyclic forms, were assessed for PS exposure after annexin V (AnV) binding. Logarithmic phase and metacyclic populations are morphologically distinct. While the former is composed of large elongated parasites with a relatively short flagellum, the latter contains parasites with a small and slightly rounded body and a very long flagellum [17], [18]. Consequently, dot plots of forward light scatter (FSC) vs. side-angle light scatter (SSC) obtained by flow cytometric analysis, clearly distinguish a large size population (FSC^HIGH^) and a small size population (FSC^LOW^) [19]. The frequency of the FSC^LOW^ population increases from 8% to 68% of the total population when parasites in the logarithmic-growth phase (Fig. 1A) progress towards the stationary-phase (Fig. 1B) of *in vitro* cultures. In the population enriched for infective metacyclics, the percentage of FSC^LOW^ parasites reaches 82% of the total population (Fig. 1C), demonstrating the high efficiency of *in vitro* metacyclogenesis and of the metacyclic purification procedure. The frequency of PS^POS^ parasites assessed by AnV binding is of 4.2% in the logarithmic population (Fig. 1D), increasing to 8.9% in the total stationary population (Fig. 1E). The frequency of PS^POS^ forms reaches 25.6% after the enrichment procedure with the monoclonal antibody 3A1La (Fig. 1F). The percentage of PS^POS^ metacyclics can reach as much as 40% of the total population, after enrichment for metacyclics at the end of the stationary phase (data not shown), fluctuating with culture conditions. Between 1% and 3% of PS^POS^ forms are found within gated FSC^HIGH^ parasites, after the purification procedure (data not shown). We confirmed PS exposure in stationary-phase and purified metacyclics by staining with an αPS monoclonal antibody (Fig. S1A). To assess if exposed PS might also play a role in natural infections, we next looked for AnV binding in metacyclic promastigotes purified from dissected guts of *Lutzomyia longipalpis* at 5 and 9 days post-infection (p. i.). As shown in Fig. 1, 13% of the promastigotes display surface PS at 5 days (Fig. 1H) and increases to 21% at 9 days after feeding (Fig. 1I). The scattered labeling pattern indicates great variability in the intensity of exposed PS within vector-derived promastigotes. The presence of 2 mM of EDTA inhibited AnV staining in vector-derived promastigotes indicating a specific annexin-PS binding (Fig. S5).

### Cooperation between PS^POS^ and PS^NEG^ forms operates in the infectivity of promastigotes

**Figure 2:**
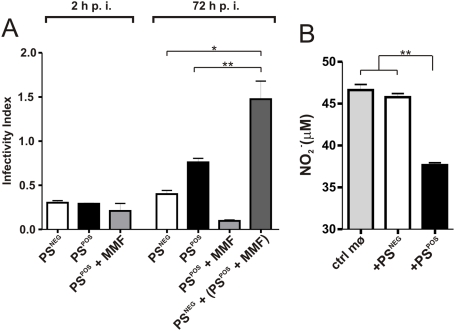
Purified PS^POS^ promastigotes are required for the *in vitro* infectivity of PS^NEG^ forms. (A) Rates of internalization (2 h p. i.) and of intracellular proliferation (72 h p. i.) of magnetic purified PS^NEG^ and PS^POS^ metacyclic promastigotes measured by their infectivity index in macrophage cultures. (B) Inhibition of NO synthesis by PS^POS^ and PS^NEG^ populations. Macrophages activated with IFNγ and LPS were infected with magnetic purified PS^NEG^ or PS^POS^ metacyclic promastigotes. After 48 h of infection, NO production by cultured macrophages were quantified by Griess reaction in the supernatants. Figure displays one experiment out of three with similar results. *p<0.001, **p<0.05.

**Figure 3:**
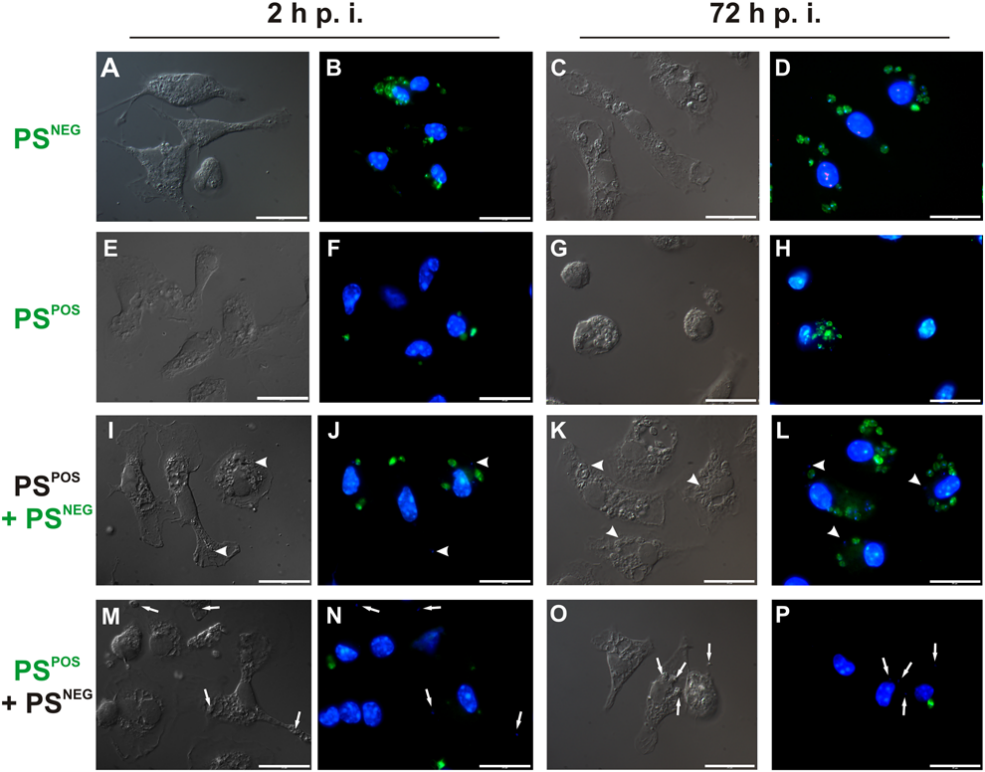
Purified PS^POS^ promastigotes are required for the *in vitro* infectivity of PS^NEG^ forms. Differential interference contrast (left panels) and fluorescence microscopy (right panels) at 2 h and 72 h p. i. of macrophage monolayers infected with CFSE-treated purified PS^POS^, PS^NEG^ and PS^POS^+PS^NEG^ (reconstituted at a 1∶1 ratio) population of promastigotes. Arrowheads indicate PS^POS^ CFSE-unlabeled parasites stained with DAPI (panels J and L). Arrows indicate PS^NEG^ CSFE-unlabeled parasites stained with DAPI (panels N and P). Nuclei are stained with DAPI. Bars represent 20 µm. Figure displays one experiment out of two with similar results.

**Figure 4:**
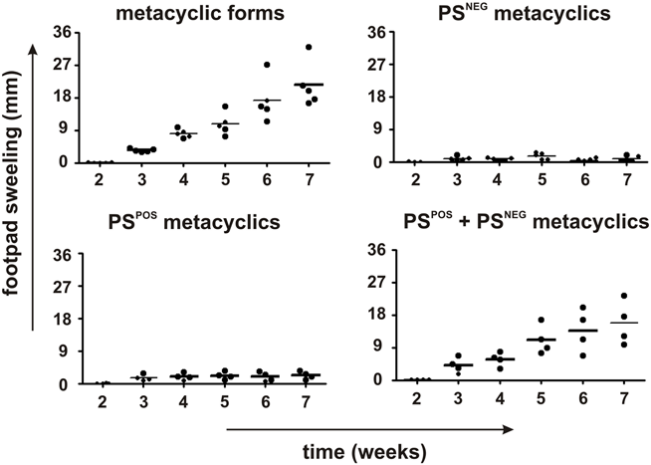
Purified PS^POS^ promastigotes are required for the *in vivo* infectivity of PS^NEG^ forms. Footpad swelling in BALB/c mice inoculated with 10^5^ metacyclic promastigotes as well as the same number of sorted PS^POS^, PS^NEG^ and PS^POS^+PS^NEG^ (reconstituted at a 1∶1 ratio) population of promastigotes. Each dot represents a single animal and the horizontal bar represents the median lesion size of each group.

To definitively show that signals provided by surface PS are required for the infectivity of promastigotes, we further purified the population enriched for metacyclic promastigotes, obtaining sub-populations of PS^POS^ and PS^NEG^ forms. Metacyclic promastigotes bound to AnV beads were submitted to magnetic cell separation, generating populations of 70–80% purity in both positive and negative fractions (Fig. S1A). For *in vitro* studies, we took advantage of the fact that promastigotes treated with mofetil mycophenolate (MMF) (an inhibitor of the purine salvage pathway) become unable to multiply after infecting macrophages, while still retaining their ability to expose PS (Fig. S1C). As shown in Fig. 2A, PS^POS^, PS^NEG^ and MMF-treated PS^POS^ populations can be equally internalized by murine macrophages after 2 h of interaction. However, at 72 h p. i. a significant increase in the number of intracellular parasites is observed only when PS^NEG^ forms are infecting macrophages in the presence of MMF-treated PS^POS^ forms, which, by themselves, are unable to multiply intracellularly (Fig. 2A). The increase in the number of parasites when the PS^POS^ population is the infective form is probably due to the number of PS^NEG^ forms contaminating this population (Fig. S1A). We evaluated nitric oxide (NO) production by activated macrophages infected with PS^POS^ or PS^NEG^ metacyclic promastigotes. PS^POS^ forms are capable of inhibiting 30% of the NO production by activated macrophages while PS^NEG^ forms do not interfere in NO production (Fig. 2B). These results were further confirmed by infecting macrophages with PS^POS^ and PS^NEG^ forms alternatively labeled with CFSE. In the Fig. 3 we clearly show that both populations can be internalized into macrophages 2 h p. i., when added either alone (Fig. 3B and F) or simultaneously (Fig. 3J and N). However, observation at 72 h p. i., clearly shows that the PS^NEG^ population is the one that multiplies intracellularly and that the number of multiplying PS^NEG^ forms is increased by the concomitant presence of PS^POS^ forms in the infecting inoculum (Fig. 3L). Interestingly, when infecting with CFSE-labeled PS^POS^ forms, an intense intracellular proliferation can be observed in a very low frequency of macrophages (Fig. 3H). In our interpretation, this is due to the presence of PS^NEG^ forms contaminating the preparation of PS^POS^ parasites. To show that this same type of cooperativeness also happens *in vivo*, we infected BALB/c mice in the hind-foot pad with 10^5^ parasites of the original metacyclic population, with the purified PS^POS^ and PS^NEG^ sub-populations, and with a population reconstituted with a 1∶1 mixture of the two previous populations, and followed lesion size in individual mice. Metacyclic PS^POS^ and PS^NEG^ promastigotes were purified by FACS sorting, with up to 90% of purity (Fig. S1B). As shown in Fig. 4, infection with the metacyclic population behaved as usual, with lesions detectable at 4 to 5 weeks p. i.; the purified populations were either unable to induce lesions up to 7 weeks p. i., or developed small non-progressive lesions. The reconstituted population behaved as the original population, with, however, some delayed lesion development. Very similar results were obtained in infected F1 (BALB/c×B6) mice (Fig. S2). However, since F1 mice are semi-resistant to leishmanial infection, lesions began to appear later and were of smaller size than in a similar experiment performed with BALB/c mice (Fig. 4).

### PS^POS^ metacyclic promastigotes are apoptotic cells

**Figure 5:**
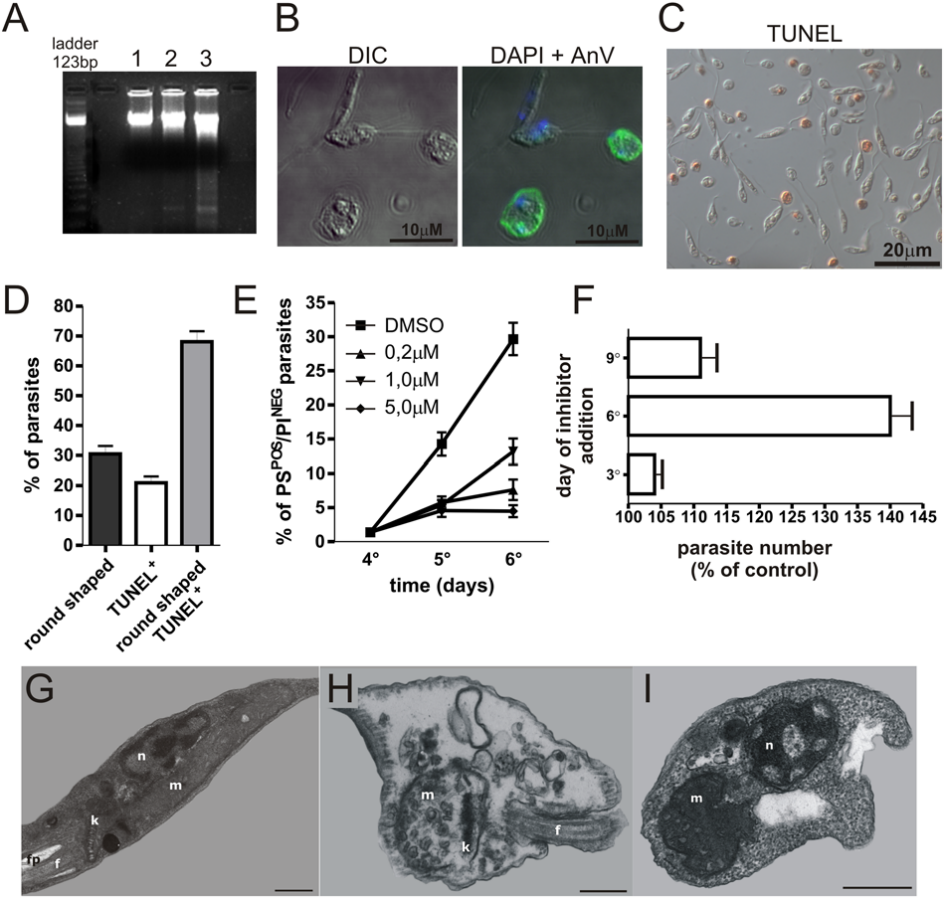
PS^POS^ parasites are apoptotic cells. (A) Oligonucleosomal DNA cleavage in logarithmic phase (lane 1), stationary phase (lane 2) and purified metacyclic (lane 3) promastigote populations. (B) Differential interference contrast (left panels) and confocal microscopy (right panels) Confocal image of purified metacyclics stained with AnV-Alexa 488 and DAPI. (C) TUNEL staining of stationary-phase promastigotes. (D) Quantification of TUNEL^POS^ and round shaped parasites by counting at least 200 parasites per slide under the microscope. (E) PS exposure by PI^NEG^ parasites treated with a pan-caspase inhibitor Z-VAD-FMK. (F) Proliferation of parasites treated with 10 µM of Z-VAD-FMK at day 3, 6 and 9 of culture. Ultrastructural analysis of (G) metacyclic promastigote and (H and I) PS^POS^ metacyclics showing morphology of nucleus (n), mitochondrion (m), kinetoplast (k), flagellar pocket (fp) and flagellum (f). Bars represent 1 µm.

Unlike amastigote forms of *La*, that despite PS exposure, are viable and highly infective [12], [13], PS^POS^ metacyclic promastigotes are non-infective, even in down-modulated macrophages. Therefore, we checked whether PS exposure by metacyclic promastigotes was due to apoptotic death. Indeed, when staining stationary-phase promastigotes with AnV-Alexa 488, it became clear that the labeled parasites are those in the process of dying or already displaying a fully roundshaped body, characteristic of dying cells, in contrast to the unlabeled elongated forms (Fig. 5B). AnV staining was found mostly at the surface of apoptotic parasites although, some intracellular stained structures could be observed (Fig. 5B and Movie S1). Those morphological characteristics were also observed by scanning electron microscopy (Fig. S4). This result was clearly confirmed by TUNEL staining. Approximatedly 20∼30% of the metacyclic population stained for TUNEL after 7 days of culture, and 70% of round shaped parasites, were TUNEL^POS^, showing a correlation between the morphology of PS^POS^ parasites and apoptotic death (Fig. 5D and Fig. S3). Furthermore, when promastigotes were treated with a pan-caspase inhibitor, Z-VAD-FMK, the number of PS^POS^ parasites decreased when the inhibitor was added on the 4th day of culture (Fig. 5E). Furthermore, promastigotes were treated with the same inhibitor at different days of culture, washed, re-seeded in new media and counted after 6 days of culture. With this protocol, an increase in surviving forms was only observed in promastigotes originally treated at day 6 of culture (Fig. 5F). This effect on parasite survival was never observed in cultures treated with the caspase inhibitor before initiation or after completion of the stationary phase of parasite growth (data not shown). In addition, we analyzed purified metacyclic forms for ultrastructural features of apoptotic death by transmission electron microscopy. As shown in figure 5G, viable parasites display an elongated cell body, the anterior flagellum emerging from the flagellar pocket, a single branched mitochondrion containing the kinetoplast with normal morphology, as well as normal nucleus morphology and chromatin condensation. In contrast, PS^POS^ parasites display several ultrastructural alterations in organelles and cytoplasm, as shown in figures 5H and I. Generally, parasites display a shorten body shape, cytoplasmic degradation, chromatin hypercondensation and disorganization of kinetoplast structure (Fig. 5H). In addition, PS^POS^ parasites exhibit a variety of morphological mitochondrial alterations, such as mitochondrial swelling, lack of matrix electrondensity and loss of the inner mitochondrial membrane organization (Fig. 5H and I). We could also observe the formation of vacuole-like structures containing parasite cytoplasmatic materials (Fig. 5H and I). Finally, oligonucleosomal DNA degradation could only be observed in the population enriched for metacyclic promastigotes, but not in logarithmic-phase promastigotes (Fig. 5A). Together, PS exposure, morphological and ultrastructural alterations, positive TUNEL staining and DNA oligonucleosomal cleavage, indicate that the fraction of metacyclic promastigotes stained with the αPS mAb or AnV is composed of parasites dying by apoptosis. The narrow time-window of sensitivity to protease inhibitors suggests that apoptotic death occurs at a defined moment of the procyclic to metacyclic transition of the promastigotes.

### Metacyclic promastigotes display ultrastructural alterations characteristic of apoptotic death and TUNEL^POS^ staining within infected sand flies

**Figure 6:**
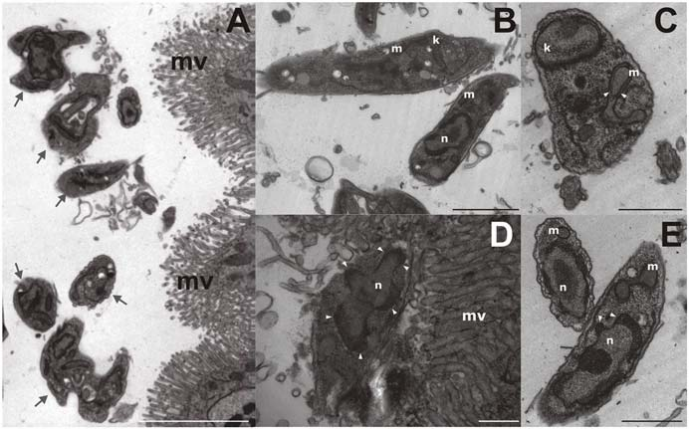
Ultrastructural analysis of metacyclic promastigotes within infected sand flies. *Lutzomyia longipalpis* sand flies were fed with heparinized mice blood containing infected macrophages. After 10 days female insects were pooled and guts were harvested and fixed for transmission electron microscopy. (A) Morphological heterogeneity of promastigotes in the intestinal lumen of a sand fly, displaying normal nucleai, kinetoplasts and mitochondria. (B) As in A with a higher magnification. (C) Promastigote showing intense mitochondrial swelling (white arrowheads) and loss of kinetoplast organization in addition to vacuole-like structures, formed by mitochondrial or endoplasmic-reticulum membranes containing cytoplasmic material. (D) Promastigote attached to intestinal microvilli showing highly condensed chromatin clumped to protrusions in nuclear lobes (white arrowheads). (E) Promastigotes with condensed chromatin and mitochondrial membrane expansions eventually containing cytoplasmic material. Bars represent 1 µm; m, mitochondrion; k, kinetoplast; n, nucleus; mv, microvili.

**Figure 7:**
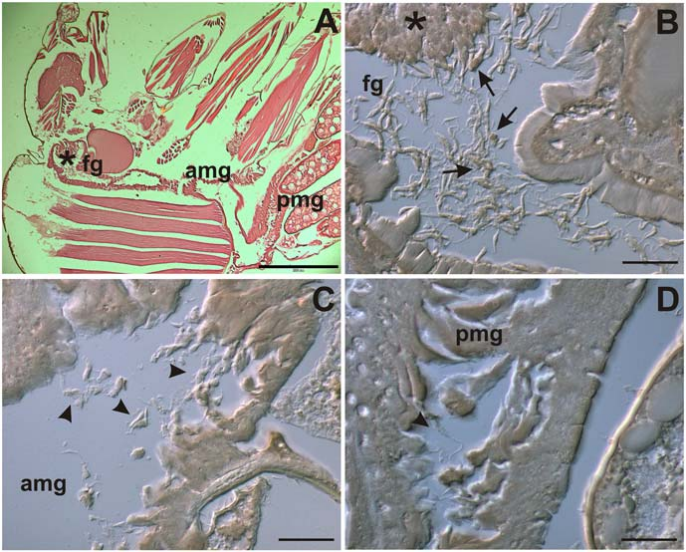
TUNEL^POS^ promastigotes are present in the intestinal tract of infected sand flies. *Lutzomyia longipalpis* sand flies were fed with mice blood containing infected macrophages. After 10 days female insects were fixed and histochemical analysis for TUNEL labeling was performed. (A) Hematoxilin-eosin stain showing overview of sand fly section. (B) Parasite concentration at the bulbous cardia region of the foregut and isolated and clumped TUNEL^POS^ promastigotes in the foregut (fg). Most of the stained parasites are already acquiring a round-shaped morphology (arrows). (C) and (D) elongated TUNEL^NEG^ promastigotes in respectively the anterior midgut (amg) and posterior midgut (pmg) of infected sand flies (arrowheads). Bars represent 200 µm (A) and 20 µm (B, C and D). Asterisks on panels A and B indicate the bulbous cardia region of the foregut.

In order to evaluate the presence of apoptotic parasites *in vivo*, we performed ultrastructural analysis of guts obtained from *Lutzomyia longipalpis* 10 days after artificial feeding. As shown in Fig. 6, parasites can be clearly distinguished in the lumen or attached to the gut wall. Viable parasites were morphologically heterogeneous, pending on the analyzed section plane, with most of them displaying normal nucleus, kinetoplast and mitochondrion. In a subpopulation of parasites, morphological abnormalities such as condensed chromatin clumped to protrusions in nuclear lobes (Fig. 6D and E), mitochondrial swelling with lack of kinetoplast organization (Fig. 6C) and vacuole-like structures, formed by mitochondrial or endoplasmic-reticulum membranes containing cytoplasmic material (Fig. 6C), were observed. These results were further confirmed by histochemical analysis of infected sand flies stained for TUNEL labeling. As shown in Fig. 7A, after 10 days of artificial feeding most of the parasites were at the bulbous cardia region of the foregut, although parasites at the posterior and anterior midgut could also be observed (Fig. 7C and D). Positive staining was clearly found in individual or clumped parasites at the foregut (fg) and anterior midgut (amg). Attached to the bulbous cardia region of the sand fly gut we could observe tightly clumped parasites that present an intense positive staining (Fig. 7B), not visualized in negative controls (Fig. S6). However, the gel-like substance that embeds this region [20] made it difficult to distinguish individual parasites. Interestingly, we did not observed TUNEL^POS^ promastigotes in parasites present at the posterior midgut and in the final part of the anterior midgut region (Fig. 7C and D). Slides stained in the absence of TdT enzyme do not show any significative brown-like staining (Fig. S6). Together these results demonstrate that PS^POS^ apoptotic parasites can be preferentially found in the anterior portion of the sand fly gut, suggesting that these cells might play a role in natural infections.

## Discussion

Apoptotic death has been described in different species of pathogenic and nonpathogenic unicellular organisms [4]. The selective advantage of such a process is still debatable and several hypotheses have been suggested [21]–[24]. We show here that, for infectivity of promastigotes of the New World species *La*, a subpopulation of the metacyclic forms undergoes apoptotic death, both *in vitro* and in the gut of a sand fly vector. As a consequence of apoptotic death, PS is exposed on the cell surface, and the interaction of this phospholipid with host macrophages plays a role in infectivity. Apoptotic death and, consequently, PS exposure occur as a consequence of the metyclogenesis process *in vivo* and *in vitro* (Fig. 1D–F and Fig. S1). In vitro, 30 to 50% of purified metacyclic parasites undergoes apoptosis, depending on culture conditions and on the day of analysis during the stationary phase of the culture. In sand fly guts, the number of PS-exposing forms, after 5 days post-feeding, is lower than that observed in axenic cultures, and the parasites are characteristically more variable in the intensity of exposed PS (Fig. 1H and I). This may be due to a more complex and less synchronized process of metacyclogenesis *in vivo* than *in vitro*. The percentage of parasites exposing PS increases after 9 days post-feeding, when most of them are already in the foregut ([19], [25] and Fig. 7F). If this increase is followed by a higher infectivity of the latter population as compared with the former one, as well as the role of apoptotic parasites on natural infections, remain to be clarified. In line with our findings, it is important to note that translocases, capable of mediating the transport of phospholipids, including PS, across the membrane of *Leishmania* promastigotes, have been described [26], [27].

The analysis of the effect of exposed PS on macrophages was evaluated *in vitro*, by sorting the total metacyclic population into PS^POS^ and PS^NEG^ subpopulations and treating the PS^POS^ cells with MMF. This inhibitor of the purine salvage pathway inhibits parasite proliferation without interfering with its ability to expose PS (Fig. S1). In this situation, non-proliferating PS^POS^ promastigotes are capable of turning logarithmic-phase promastigotes (data not shown) or PS^NEG^ parasites (Fig. 2A) into infective populations. This is probably due to the capacity of the PS^POS^ promastigotes to inhibit NO production by activated macrophages (Fig. 2B), similarly to that occurs with PS on the surface of amastigotes [12], [13]. The fact that the PS^POS^ forms are only signaling parasites while PS^NEG^ forms are the ones that differentiate into amastigotes and multiply inside macrophages, was further confirmed by labeling PS^POS^ and PS^NEG^ parasites with CFSE and following their fate inside macrophages (Fig. 4). The higher load of intracellular labeled amastigotes is attained when macrophages are infected with a 1∶1 combination of labeled PS^NEG^ parasites and unlabelled PS^POS^ forms, and infection is allowed to proceed for 72 h (Fig. 3L). On the contrary, when the PS^POS^ population is the labeled one, very few labeled parasites can be observed after 72 h p. i. (Fig. 3P). One interesting finding is that at 72 h p. i. with the labeled PS^POS^ population, a low frequency of sparsely distributed and highly infected macrophages can be observed (Fig. 3H). In our interpretation few contaminating PS^NEG^ parasites (Fig. S1), generates highly infected macrophages which are exquisitely inactivated by the high number of PS^POS^ forms present in the infective inoculum. Cooperativeness between PS^POS^ and PS^NEG^ forms was definitely demonstrated by the *in vivo* evidence that none of the purified populations was able to progress and generate a lesion in susceptible (Fig. 5) and semi-resistant mice (Fig. S2); only a reconstituted population was capable of inducing lesions similar to those obtained with the original metacyclic population. Altruistic apoptotic death was hypothesized before [19], [28], [29] and these findings, allow us to consider apoptotic death of metacyclic promastigotes as a stable altruistic behavior in the context of a host parasite interaction, as already described on yeast populations [30], [31].

As a matter of fact, taking advantage of the anti-inflammatory properties of exposed PS seems to be an adaptive strategy of trypanosomatids and intracellular parasitic organisms in general. This can occur by self-exposing this moiety or by using host-cell apoptosis for their own benefit. Indeed, *Trypanosoma cruzi*, besides exposing PS in its infective form [32], induces apoptotic host T-cells for macrophage inactivation and consequent persistence in the mammalian host [33]. *L. major* promastigotes survives inside neutrophils which, by becoming apoptotic, are ingested by macrophages, and after ingestion, neutrophils release viable parasites in an already inactivated host cell [34]. Interestingly, the interaction with apoptotic, but not with necrotic neutrophils, facilitates infection of human macrophages with *La* [35]. Infective tachyzoites of *T. gondii*, by exposing PS, inhibit iNOS and NFκB activation on infected macrophages, via a TGFβ-dependent mechanism [36]. PS exposure usage as a way to infect and reside in host cells is not restrict to parasitic protozoa. Recent data demonstrated that different viruses, such as vaccinia, cytomegalovirus and Lassa fever virus employ similar strategy to infect host cells [14], [37]. These reports lead to the development of an antiviral therapy that enhance cytotoxicity of viral-infected cells, based on an αPS specific antibody [37].

The evidence that PS exposure by a sub-population of metacyclic promastigotes is a step of an ongoing process of apoptotic death is shown by DNA cleavage, TUNEL staining, morphological and biochemical features (Fig. 5 A–D, Fig. S4 and Movie S1). Most surprisingly, adding caspase inhibitors at the beginning of the stationary-phase significantly reduces the number of apoptotic promastigotes, as assessed by a decrease in the number of PS^POS^ forms (Fig. 6E), and of parasites dying at the stationary-phase of the culture (Fig. 6F). The nature of the molecules involved in these latter results is still under scrutiny. In spite of reports of caspase-like activities in *Leishmania spp* [38]–[40], no orthologous caspase sequences were identified in the genomes of trypanosomatids and it is also still unclear whether metacaspases are involved in apoptotic death [41]. Contrary to convincing evidences showing caspase-like activities of metacaspases from plants and fungi [42], [43], protozoan metacaspases seem to preferentially cleave trypsin-like substrates [44] and play a role in cell cycle control [45]. Our results however indicate that at a very specific time-point of the promastigote cell cycle (logarithmic to stationary phase transition), promastigote survival can be modulated by caspase inhibitors. It is tempting to speculate that during this narrow time-window, apoptotic death dependent on caspase-like activation can be operational and of biological importance. This hypothesis, as well as the eventual involvement of metacaspases, remains to be proven.

Furthermore, in both, *in vitro* (Fig. 5E–G) and *in vivo* (Fig. 7C–E) a population of promastigotes displays a variety of ultrastructural markers not only of apoptotic but also of autophagic death, a process described as vital for *Leishmania* differentiation [46]–[48]. It is thus possible that some of these structures represent autophagic death occurring during metacyclogenesis. Since autophagic and apoptotic cell death mechanisms can be redundant, our findings may reflect both processes occurring simultaneously, or alternatively, one leading to the other as postulated by Golstein and Kroemer, [49]. The fact that PS exposure has been detected in the gut of sand flies and that TUNEL^POS^ metacyclic parasites accumulate at the bulbous cardia region of the foregut (Fig. 6F–I), strongly suggests that apoptotic death in the gut of the vector is part of the *in vivo* metacyclogenic differentiation and plays a role in natural infections. Since PS exposure alone, as reported with *L. major* promastigotes [16], is not enough to indicate apoptosis, the ultrastructural, spatial and biochemical characterization of parasites in the gut of sand flies provide solid data to indicate the occurrence of apoptotic parasites *in vivo*. The presence of apoptotic parasites in the vector gut, in addition to several other elements that interferes with vector competence [50] is certainly a prerequisite for their participation in the infective process. If they are inoculated in the mammalian host as non-infected altruistic forms [51] remains to to be shown. We have previously shown that heat-shock (34°C to 37°C) induces apoptotic death in promastigotes [5], with ultrastructural features similar to the ones found in the present paper. It is thus tempting to propose that apoptotic death progresses during metacyclogenesis in the vector and that due to the temperature change occurring during insect to mammalian host passage, the number of PS-exposing promastigotes increases at the inoculation site.

## Materials and Methods

### Mice

BALB/c and F1(BALB/c×C57BL6) mice were used. Animal handling in our facility complies with the International Guiding Principles for Biomedical Research Involving Animals and is approved by the Brazilian Committee for Animal Experimentation (COBEA).

### Cells

*La* (MPRO/BR/1972/M-1841) promastigotes maintained by a maximum of six sequential *in vitro* passages were used. To obtain a pure population of logarithmic-phase promastigotes, cultures were replicated every 2 days for at least 3 consecutive cycles. Metacyclic promastigotes were purified from stationaryphase cultures by negative selection using the 3A1-*La* monoclonal antibody [52] specific for procyclic (non-infective) forms.

### Flow cytometric analysis, confocal and fluorescence microscopy

Parasites were washed, suspended in AnV binding buffer (ABB - HEPES 10 mM, NaCl 150 mM, CaCl_2_ 2.5 mM) at pH 7.2. Cells were incubated at room temperature for 15 min with AnV-FITC (Molecular Probes, Eugene, OR, USA) at the concentration indicated by the manufacturer. At the moment of acquisition, 0.4 µg/ml of propidium iodide (PI) were added to control and AnV-FITC-labeled samples. Data were collected in a BD FACSCalibur® and analyzed by Cellquest Pro® (BD Biosciences, San Jose, CA, USA). Ten thousand gated events were harvested from each sample. For confocal microscopy, parasites were incubated for 30 min, 4°C with 500 µg/ml of AnV-Alexa 488 (MolecularProbes, Eugene, OR, USA) in ABB. As a control, the same procedure was followed in the presence of 2 mM of EGTA. Hence, parasites were adhered in poly-L-lysine-treated coverslips and slides were mounted with Vectashield Mounting Medium with DAPI (Vector Laboratories, Burlingame, CA, USA). Mounted slides were visualized by using a Zeiss LSM 510 UV META Laser Scanning Confocal Microscope.

### Purification of PS^POS^ and PS^NEG^ promastigotes

Metacyclic promastigotes obtained from stationary-phase culture were labeled with AnV-FITC (Molecular Probes, Eugene, OR, USA) as described above. Flow cytometric sorting was performed using a BD FacsAria® (BD Biosciences, San Jose, CA, USA). Prior to infection, parasites were treated for 5 min at 4°C with PBS containing 2 mM of EGTA to detach AnV-FITC from the parasite surface. Parasites were washed once with PBS containing 2 mM of EGTA and suspended in complete medium. Alternatively, magnetic cell separation of PS^POS^ and PS^NEG^ promastigotes was performed. To this end, metacyclic promastigotes were separated using AnV Microbead Kit (Miltenyi Biotec Inc. Auburn, CA, USA).

### Sand fly infection and parasite purification

J774 macrophages were infected with stationary-phase *La* promastigotes. After 48 h of infection, cells were harvested and suspended in heparinized BALB/c mouse blood. This suspension was used to feed *Lutzomyia longipalpis* sand flies through a chick skin membrane. At different days p. i., female sand flies were pooled for gut dissection and infection evaluation by optical microscopy. Infected guts were slit open in PBS and parasites spontaneously moving outwards, were collected for analysis.

### Macrophage infection and nitric oxide determination

Thyoglicolate-elicited peritoneal macrophages collected from BALB/c mice were plated and non-adherent cells were removed by washing in Hank's Balanced Salt Solution (HBSS - Sigma-Aldrich Co, St. Louis, MO, USA) after 2 h incubation at 37°C. MMF-treated (1 mM for 30 min) PS^POS^ parasites, PS^NEG^ or PS^POS^ promastigotes were added to adhered macrophages, at a 3∶1 ratio. After 2 h incubation at 34°C, free parasites were removed by extensive washing and cultures proceeded for additional 2, 48 or 72 h p. i. The infectivity index (percentage of infected macrophages×average number of amastigotes per macrophage) was calculated by randomly counting at least 200 macrophages per slide. For NO synthesis determination, peritoneal macrophages cultures were infected and stimulated with 100 ηg/ml of murine interferon-γ. (Peprotech Inc. New Jersey, USA) and 100 ηg/ml of LPS (Sigma-Aldrich Co, St. Louis, MO, USA) for 48 h. Supernatants were assessed for nitric oxide concentration by Griess reaction (Sigma) and compared with a standard NaNO_2_ curve.

### CFSE labeling of promastigotes

Parasites were suspended in PBS containing 5 µM of CFSE (Molecular Probes, Eugene, OR, USA) and incubated at room temperature for 5 min. Labeled parasites were washed four times with PBS and suspended in culture medium before addition to macrophage cultures.

### *In vivo* infections

BALB/c or F1(BALB/c×C57BL6) mice were sub-cutaneously infected into hind footpad with 10^5^ parasites as indicated. Footpads were measured weekly by using a direct reading Vernier caliper. The results are expressed as footpad area (length×width).

### Ultrastructural analysis

Stationary-phase promastigote forms were fixed overnight in 2.5% glutaraldehyde, 4% of sucrose (w/v), 4% of paraformaldehyde and 5 mM of CaCl_2_ in 0.1 M of cacodylate buffer, and post-fixed with 1% osmium tetroxide and 0.8% potassium ferrocyanide in 0.1 M of cacodylate buffer for 1 h. Parasites were washed in the same buffer, dehydrated in acetone and embedded in Epon resin. Guts of infected sand flies were collected and the same protocol as above was used.

### Caspase-inhibitor treatment

Parasites were incubated in complete Schneider's Insect Medium with 10 µM of Z-VAD-FMK (Sigma-Aldrich Co, St. Louis, MO, USA) at 3, 6 and 9 days of culture respectively at the logarithmic, early and late stationary phases. After 1 h incubation at 22°C, the cells were washed twice and the culture was left to proceed at the same conditions in complete medium. The number of cells was measured after 6 days of culture by the MTT assay and by counting in a Neubauer chamber. For PS exposure experiments, parasites were cultured in complete medium and after 4 days of culture, Z-VAD-FMK was added at the indicated concentrations. The percentage of PS^POS^/PI^NEG^ parasites was evaluated by flow cytometry at the indicated time points.

### TUNEL staining

Promastigote forms were fixed in 4% paraformaldehyde and adhered in poly-L-lysine-treated coverslips overnight. Parasites were labeled for TUNEL staining with the DeadEndTM Colorimetric TUNEL System (Promega Madison, WI, USA) following manufacturer instructions. Infected sand flies were harvested, incubated in ice-cold PBS, fixed with 2.5% glutaraldehyde, 4% of sucrose (w/v), 4% of paraformaldehyde and 5 mM of CaCl_2_ in 0.1 M of cacodylate buffer and embedded in paraffin. Sections of 4–5 µM were mounted onto slides and evaluated for TUNEL staining.

### Statistical analysis

Unpaired t test with Welch correction (*in vitro* assays) and a one way analysis of variance (ANOVA) followed by the Bonferroni multiple comparison test (*in vivo* assays) were used to compare differences between means of replicates of different experimental points.

## Supporting Information

Figure S1 (0.48 MB TIF)

Figure S2 (0.24 MB TIF)

Figure S3 (0.77 MB TIF)

Figure S4 (0.82 MB TIF)

Figure S5 (0.39 MB TIF)

Figure S6 (1.87 MB TIF)

Movie S1 (0.17 MB MOV)
